# Comparative proteomic analysis of cancer stem cells in a Tax-transgenic (Tax-Tg) mouse model of adult T-cell leukemia/lymphoma

**DOI:** 10.1186/1742-4690-8-S1-A9

**Published:** 2011-06-06

**Authors:** Tadaki Suzuki, Akiko Okayama, Takahiro Tsuji, Akihide Ryo, Hisashi Hirano, Tetsutaro Sata, William W Hall, Hideki Hasegawa

**Affiliations:** 1 Department pathology, National Institute of Infectious Diseases, Musashimurayama, 208-0011, Japan; 2 Advanced Medical Research Center, Yokohama City University, Yokohama, Japan; 3 Department of Microbiology and Molecular Biodefense Resarch, Yokohama City University, Yokohama, Japan; 4 Center for Research in Infectious Disease, University College Dublin, Dublin, Ireland

## 

Adult T-cell leukemia (ATL) is a T-cell malignancy caused by HTLV-1. In ATL, chemotherapeutic responses are generally poor, which has suggested the existence of chemotherapy-resistant cancer stem cells (CSCs). The HTLV-I transactivator, Tax, initiates ATL-like disease (mATL) in Tax transgenic mice. Recently, the CSCs of mATL were identified. The CSCs in a side population, could consistently regenerate the original lymphoma and overlapped with a minor population of CD38(-)/CD71(-)/CD117(+) cells, suggesting that the eradication of cancer stem cells will be necessary to improve the outcome of treatment for mATL. In designing specific regimens for CSCs, antibody-based therapy appears to be a promising way to destroy CSCs. A small number of target antigens on cancer stem cells have been described. It remains to be determined, however, whether these and other targets will distinguish cancer stem cells from normal tissues.

In this study, a proteomic approach was used to identify proteins differentially expressed in CSCs of mATL. The CSCs of mATL were isolated using cell sorting, and analyzed by label-free LC-MS/MS to compare protein profiles in CSCs and no-CSCs. More than 700 proteins were detected. The levels of 53 proteins were increased in CSCs. An interesting finding is that these proteins included one membrane protein, which might potentially serve as a new target of antibody-based therapy. Flow cytometry validated expression of the membrane protein. Taken together, the data presented provide a significant new protein-level insight into the biology of cancer stem cells of mATL, a key population which are involved in mATL development.

